# Supplementary material for: Spatial Variation in Stream-Water Bacterial Communities Across Sites Differing in Wild Chinese Giant Salamander Detection Frequency
Source: Biology (Basel). 2026 Sep 18;15(18):1647. doi: 10.3390/biology15181647 (PMC13604818; doi:10.3390/biology15181647)
Supplement: Supplementary file 1 [file biology-15-01647-s001.zip › Data Related to “Spatial Variation in Stream-Water Bacterial Communities across Sites Differing in Wild Chinese Giant Salamander Detection Frequency”/Microbial Data/result/03.TaxonVisual/heatmap/sample/heatmap.sample.c.cluster.pdf]

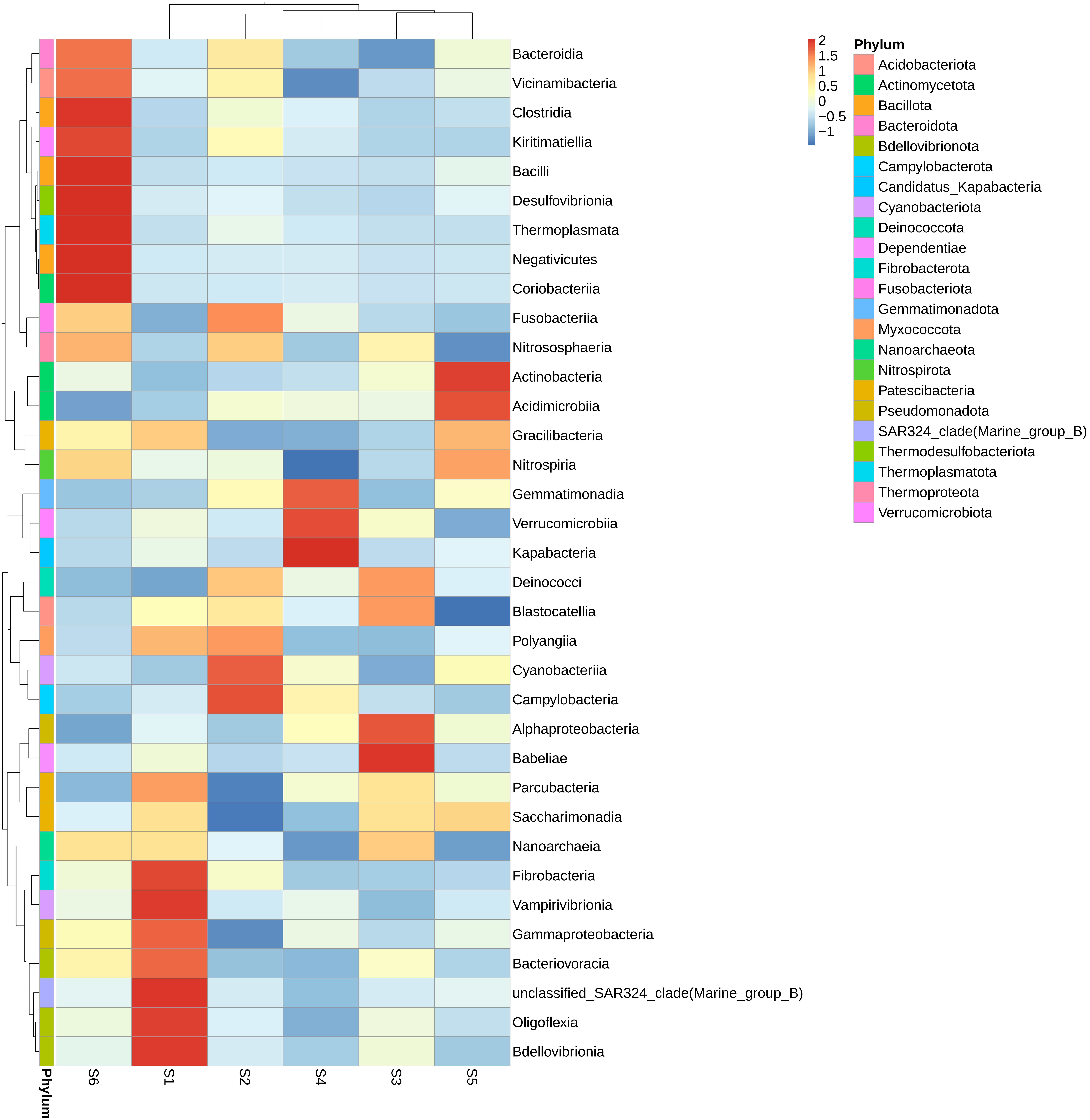

Bacteroidia
Vicinamibacteria
Clostridia
Kiritimatiellia
Bacilli
Desulfovibrionia
Thermoplasmata
Negativicutes
Coriobacteriia
Fusobacteriia
Nitrososphaeria
Actinobacteria
Acidimicrobiia
Gracilibacteria
Nitrospiria
Gemmatimonadia
Verrucomicrobiia
Kapabacteria
Deinococci
Blastocatellia
Polyangiia
Cyanobacteriia
Campylobacteria
Alphaproteobacteria
Babeliae
Parcubacteria
Saccharimonadia
Nanoarchaeia
Fibrobacteria
Vampirivibrionia
Gammaproteobacteria
Bacteriovoracia
unclassified_SAR324_clade(Marine_group_B)
Oligoflexia
Bdellovibrionia
Phylum
S6
S1
S2
S4
S3
S5
2
1.5
1
0.5
0
−0.5
−1
Phylum
Acidobacteriota
Actinomycetota
Bacillota
Bacteroidota
Bdellovibrionota
Campylobacterota
Candidatus_Kapabacteria
Cyanobacteriota
Deinococcota
Dependentiae
Fibrobacterota
Fusobacteriota
Gemmatimonadota
Myxococcota
Nanoarchaeota
Nitrospirota
Patescibacteria
Pseudomonadota
SAR324_clade(Marine_group_B)
Thermodesulfobacteriota
Thermoplasmatota
Thermoproteota
Verrucomicrobiota
